# The well-coordinated linkage between acidogenicity and aciduricity via insoluble glucans on the surface of *Streptococcus mutans*

**DOI:** 10.1038/srep18015

**Published:** 2015-12-10

**Authors:** Lihong Guo, Jeffrey S. McLean, Renate Lux, Xuesong He, Wenyuan Shi

**Affiliations:** 1 School of Dentistry, University of California, Los Angeles, CA90095, USA; 2 Department of Periodontics, University of Washington, Seattle, WA, 98195, USA; 3 J. Crag Venter Institute, La Jolla, CA 92037, USA; 4 C3-Jian Inc. Marina del Ray, CA 90292 USA

## Abstract

*Streptococcus mutans* is considered the principal cariogenic bacterium for dental caries. Despite the recognition of their importance for cariogenesis, the possible coordination among *S. mutans’* main virulence factors, including glucan production, acidogenicity and aciduricity, has been less well studied. In the present study, using *S. mutans* strains with surface-displayed pH-sensitive pHluorin, we revealed sucrose availability- and Gtf functionality-dependent proton accumulation on *S. mutans* surface. Consistent with this, using a pH-sensitive dye, we demonstrated that both *in vivo* cell-produced and *in vitro* enzymatically synthesized insoluble glucans displayed proton-concentrating ability. Global transcriptomics revealed proton accumulation triggers the up-regulation of genes encoding functions involved in acid tolerance response in a glucan-dependent manner. Our data suggested that this proton enrichment around *S. mutans* could pre-condition the bacterium for acid-stress. Consistent with this hypothesis, we found *S. mutans* strains defective in glucan production were more acid sensitive. Our study revealed for the first time that insoluble glucans is likely an essential factor linking acidogenicity with aciduricity. The coordination of these key virulence factors could provide new insights on how *S. mutans* may have become a major cariogenic pathogen.

Dental caries is one of the most prevalent bacteria-related infectious diseases worldwide^1^,^2^. The primary etiological factor of caries is acid production from dietary carbohydrates by the cariogenic bacteria in dental biofilms^3^. As the principle causative organism of dental caries, *Streptococcus mutans* possesses many physiological traits relevant to its cariogenesis^3^. By rapid fermentation of carbohydrates, *S. mutans* is able to generate acidic end products (acidogenicity) which is not only the direct causative factor for demineralization of tooth surfaces, but also an environmental determinant that may impact the caries-related microbial flora during cariogenic process^4^. Meanwhile, *S. mutans* has also developed adaptive acid tolerance response (ATR) to combat the destructive nature of an acidic environment it produces (aciduricity)^5^. It is characterized by the induction of multiple cellular pathways upon exposure to mildly acidic conditions to allow cells to better adapt to acid challenge. For example, for maintenance of intracellular pH homeostasis, *S. mutans* alters its membrane composition to prevent proton from influx and increases proton extrusion via end-product efflux and acid-stable proton-translocating F_1_F_0_-H/ATPase activity^6^,^7^,^8^. *S. mutans* is able to alter its metabolic activity through enhancing glycolytic activity and synthesis of branched amino acids during acid stress^9^,^10^. It also up-regulates the expression of variety of chaperons, such as DnaK and DnaJ to protect and repair macromolecule damage due to the harmful effects of intracellular acidification^5^.

Another important virulence factor contributing to *S. mutans’* cariogenecity is glucan, particularly insoluble glucan production^11^. In a dental biofilm, 10–20% of the dry weight is composed of EPS^12^ most of which is glucans synthesized by microbial glucosyltransferases (Gtfs) from substrate sucrose^13^. *S. mutans* is a major contributor to the production of glucans in dental biofilm^14^. In *S. mutans*, the synthesis of extracellular glucans from sucrose is catalyzed by three GTF enzymes. GtfB synthesizes mostly water-insoluble glucans; GtfC catalyzes the synthesis of a mixture of water-insoluble and soluble glucans; whereas GtfD exclusively synthesizes water-soluble glucans^15^,^16^. The insoluble glucans synthesized by surface-adsorbed GtfB and GtfC provide binding sites for the establishment of *S. mutans* on the solid surface as well as facilitate its coadherence with other bacterial cells^17^. It plays essential role in mediating the transition from initial cell attachment and clustering to microcolony formation and multi-microcolonies aggregates^17^. Deletion of both *gtfB* and *gtfC* caused the maximum reduction in *S. mutans* extracellular polysaccharide matrix production and biofilm formation, as well as decreased incidence of smooth surface caries in a rat model^18^ .

In addition to facilitating the biofilm formation, glucans have also been proposed to act as diffusion barrier and increase the sieving effect of dental biofilm, which could trap acid near the tooth surface and contribute to the demineralization process^19^. In a recent study, by using a surface-displayed pH sensitive green fluorescent protein (pHluorins), we revealed a much lower and well-maintained pH on cell surface than micro-environment surrounding the cells, suggesting the potential involvement of surface-associated component in influencing the accumulation of protons on cell surface^20^. Meanwhile, the frequent sucrose intake and microenvironmental acidification has been shown to induce *gtf* gene expression and enable *S. mutans* to accumulate glucans continuously under low pH conditions^21^. Taken together, these data suggested a possible interplay among acid production, acid tolerance and the glucans synthesis. In this study, we revealed an additional important role of glucans in cariogenic process by linking acidogenicity and aciduricity, two of the main virulence factors of *S. mutans*. Our data showed that *S. mutans* is able to concentrate protons via surface-associated insoluble glucans. The resultant low surface pH triggers ATR and allows *S. mutans* to better adapt to acid stress.

## Results

### Sucrose availability- and Gtf functionality-dependent proton accumulation on *S. mutans* cell surface

**Figure 1:**
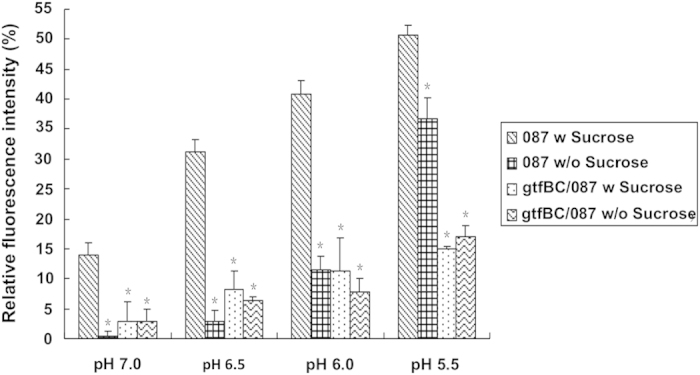
Percentage changes of fluorescence intensity in *S. mutans* wild-type and *gtfBC*-deficient strains upon switching to different pH values. *S. mutans* strain O87 [O87] and its *gtfBC*-deficient strain [gtfBC/O87] were pre-grown in buffered pH7.5 medium and switched to the indicated pH values. The fluorescence signal reduction was calculated as the percentage of signal reduction relative to the initial signal at pH7.5. The plots show the average of triplicate samples, and the error bars correspond to the standard deviations. Student’s *t*-test was used to calculate the significance of the difference between the strain O87 supplemented with sucrose and that supplemented with glucose, and also between the *gtfBC* mutant and its parent strain. *Star indicates statistical significance between the value of indicated sample and that of strain O87 subjected to the same treatment (p < 0.05).

In a recent study, pH was monitored on the cell surface of *S. mutans* O87, a strain with cell surface-displayed pH sensitive green fluorescent protein (pHluorins), whose fluorescence signal intensity reduced corresponding to the decrease in surrounding pH^20^. The observation that a well-maintained, lower pH was monitored on the cell surface than in the macro-environment suggested the potential involvement of *S. mutans* surface-associated component, likely insoluble glucans in concentrating protons on cell surface. To further test this hypothesis, surface-displayed pHluorins was used to monitor the changes of pH value on the cell surface when *S. mutans* O87 cells were exposed to the medium with different pH values in a real time, *in situ* manner. The strain O87 cells grown in buffered minimal defined medium (MDM) (pH 7.5) in the presence of sucrose or glucose were switched to buffers with lower pH values (pH 7.0, 6.5, 6.0 and 5.5) and their surface fluorescence intensity was monitored after 10 minutes’ incubation. As shown in Fig. 1, when switched from pH 7.5 to each buffer with lower pH value, *S. mutans* cells pre-grown in the presence of sucrose exhibited significantly more reduction (p < 0.05) in the surface fluorescence intensity, indicating a greater cell surface pH drop compared with the cells grown in the presence of glucose. Meanwhile, cells experienced the most drastic surface pH drop (as indicated by more severe reduction in fluorescence signal) when external pH shifted from 7.5 to 5.5, suggesting that there existed a dose-effect relationship between proton amounts in surrounding environment and pH drop on the *S. mutans* surface.

To further determine if GtfBC activity is required for the pH reduction on the cell surface, a mutant deficient in *gtfBC* while carrying cell surface-displayed ecliptic pHluorin was used in a similar experimental setup. Result showed that, in contrast to parent strain O87, GtfBC-defective strain grown in the presence or absence of sucrose exhibited a significantly less change (p < 0.05) in fluorescence intensity when switched from pH 7.5 buffer to each tested buffer with lower pH value (Fig. 1). These data showed that the pH reduction on the cell surface is associated with *S. mutans’* GtfBC activity and dependent on the availability of sucrose.

### *S. mutans* accumulates protons on cell surface by synthesized insoluble glucans

The above data showed that the observed pH reduction on the cell surface of *S. mutans* is dependent on both the availability of sucrose and the functionality of GtfBC. Since GtfB and GtfC are enzymes responsible for the production of insoluble glucans using sucrose as substrate^13^ it is likely that the surface-associated insoluble glucans synthesized by *S. mutans* GtfBC were responsible for the accumulation of protons and reducing cell surface pH value. To address this, we utilized pH-sensitive pHrodo Green dye, whose fluorescence signal intensity increases corresponding to the decrease in surrounding pH, to test the ability of surface-associated insoluble glucans to accumulate protons.

**Figure 2:**
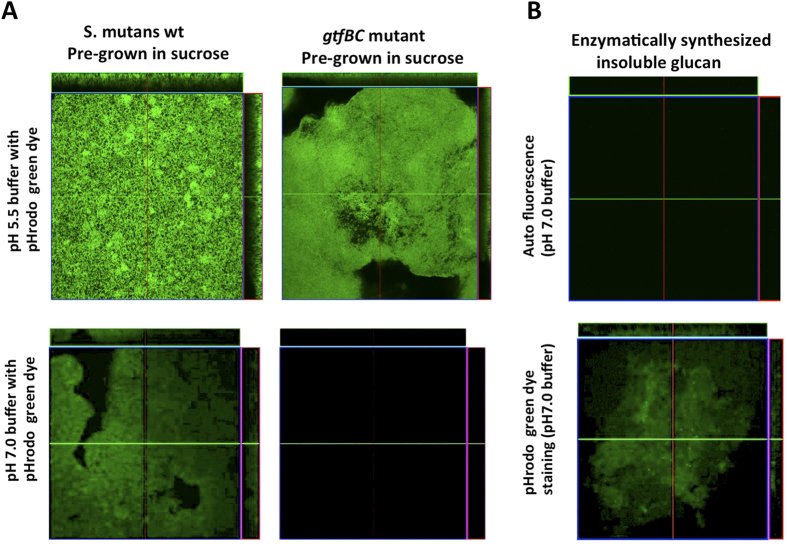
pHrodo Green STP Ester staining of *S. mutans* cells and *in vitro* prepared insoluble glucans. (**A**) *S. mutans* UA 140 wildtype or gtfBC mutant cells pre-grown in the buffered (pH7.0) MDM supplemented with sucrose (20 mM) were harvested and stained with in pHrodo green dye in buffers with different pH (pH 5.5 and 7.0); and (**B**) *in vitro* prepared insoluble glucans were stained by pHrodo Green STP Ester in pH7 buffer.

Results showed that when *S. mutans* wildtype or *gtfBC* mutant pre-grown in the buffered MDM (pH7.0) supplemented with sucrose was submerged in pH 5.5 buffer containing pHrodo Green dye, cells displayed bright fluorescence signal (Fig. 2A). However, when immersed in dye-containing pH 7.0 solution, wild type cells pre-grown in the presence of sucrose still displayed detectable green fluorescence signal, while no signal was observed for similarly cultured *gtfBC* mutant. Our data further implicated the involvement of surface-associated insoluble glucans in accumulating protons and achieved a cell surface pH lower than that in surrounding medium.

To more directly determine if cell’s ability to concentrate protons is due to the presence of insoluble glucans on the cell surface, Gtfs were extracted and Gtf-derived insoluble glucans were prepared *in vitro* to test their ability to accumulate protons using pH-sensitive pHrodo Green dye. As shown in Fig. 2B, glucans did not display auto-fluorescence when immersed in pH 7.0 buffer. When incubated in pH 7.0 buffer containing pHrodo Green dye, the insoluble glucans pellets showed significant amount of fluorescence signal, indicating insoluble glucans were able to accumulate protons and decrease cell surface pH lower than that of the surrounding buffer (pH 7.0).

### Low pH and insoluble glucans-dependent differential gene expression in *S. mutans*

Our data showed that insoluble glucans are able to concentrate protons, which could potentially precondition *S. mutans* against acid insults by inducing cellular acid-tolerance response. To test this, we further investigated the low pH-induced changes in the transcriptional profiles of *S. mutans* wild-type and *gtfBC* mutant cells pre-grown in the presence of sucrose or glucose using RNA-seq.

**Figure 3:**
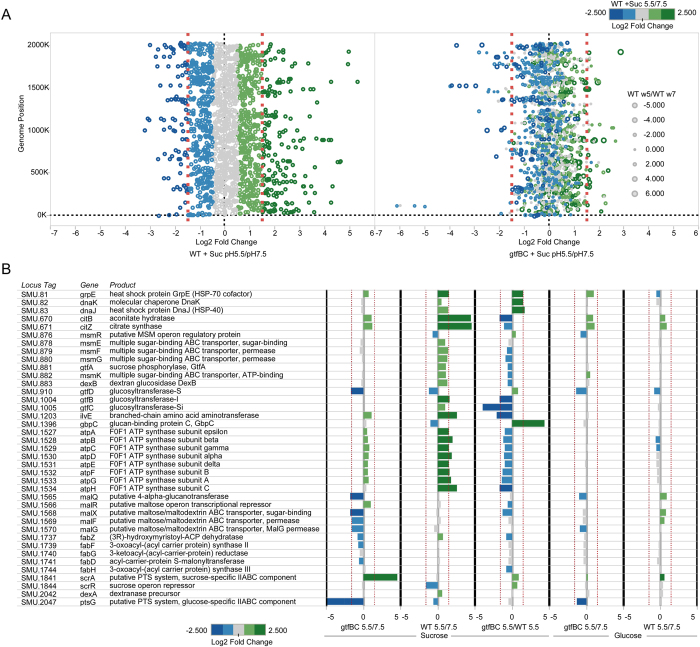
Global transcriptome profiling and differentially expressed genes previously observed in Acid Tolerance Response (ATR) and sugar metabolism in WT and gtfBC mutant detected through RNAseq. (**A**) Differentially expressed genes between pH 5.5 and pH 7.5 across the genome in WT (upper left panel) and gtfBC deficient stain (upper right panel). Expression ratios are log2 fold changes. Expressed genes represented by open circles are colored and sized based on the log_2_ fold change in expression between WT at pH 5.5 and pH 7.5. Gene expression changes in the gtfBC mutant that show similar or opposing trends as the WT are then visible in the plot. (**B**) Differential expression of select key genes previously associated with ATR as well as sugar metabolism pathways and their log_2_ fold changes. Red dashed vertical lines indicate significant fold change.

Our transcriptomic analysis revealed roughly 200 genes that were differentially regulated when wildtype *S. mutans* pregrown in buffered pH 7.0 MDM supplemented with sucrose was exposed to pH5.5 compared to pH7.5 solution; while this number reduced to about 100 when *gtfBC* mutant was tested (Fig. 3A, Figs S1–S4)(Table S2). In the wild-type background, the exposure of *S. mutans* cells pre-grown in the buffered (pH7.0), sucrose-containing MDM to pH5.5 solution induced the expression change of a multitude of genes spanning the whole genome and encoding diverse functions (Fig. 3B) (Fig. S1, S2). Many of the up-regulated genes observed in the WT have been implicated in acid tolerance response (Fig. 3), including *msm* and *atp* operon, which encode multiple sugar metabolism (MSM) transport system and F_1_F_0_-H/ATPase, as well as genes encoding chaperons (grpE, dnaK and dnaJ) and enzymes (aconitate hydratase and citrate synthase) involved in citrate metabolism. Most intriguingly, these genes were either not responsive or only induced to a very low level in the *gtfBC* background when cells were exposed to the same low pH (Fig. 3B) (Fig. S1). Meanwhile, the *gtfB* and *gtfC* genes were also up-regulated in response to lower pH in a similar *gtfBC* dependent manner. Furthermore, a subset of genes encoding functions such as fatty acid biosynthesis (*fab* operon) and putative maltose/maltodextrin ABC transporter (*mal* operon) did not respond to the low pH in the wildtype background, but displayed reduced expression in *gtfBC* mutant when exposed to same low pH. To further confirm the transcriptome data, real-time quantitative PCR was performed to monitor the expression of a panel of selected genes, including two of the F_1_F_0_-H/ATPase subunit-encoding genes, *atpA* and *atpD*; two multiple sugar binding ABC transporter component-encoding genes, *msmK and dexB*; as well as *gtfB* and *gtfC*. Overall, the qRT-PCR results were in agreement with our transcriptome findings (Table S3).

### Insoluble glucan-dependent acid tolerance in *S. mutans*

**Figure 4:**
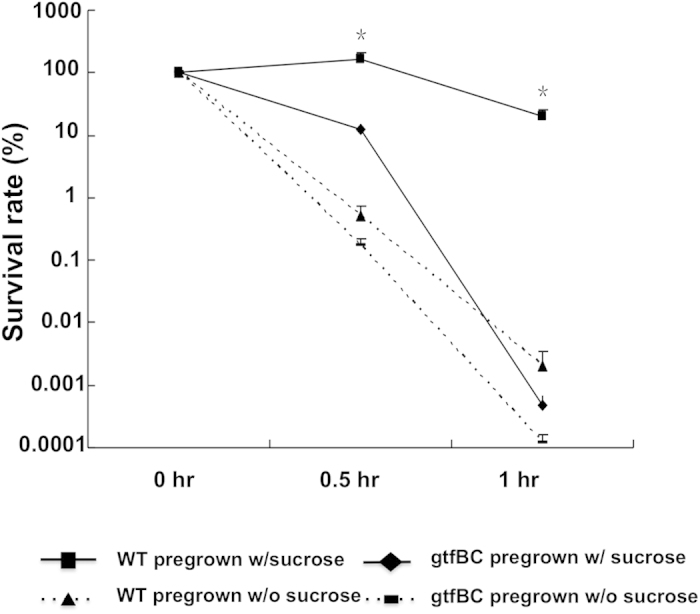
Acid killing assay of *S. mutans* wt and gtfBC strains pre-grown the presence or absence of sucrose. Cells of *S. mutans* wild-type [WT] and *gtfBC*-deficient strain [gtfBC] were pre-grown in the buffered minimal medium (pH 7.5) supplemented with glucose or sucrose (20 mM). The cultures were then rapidly acidified to pH 2.8 using glycine solution, and incubated for 0, 0.5 h and 1 h, respectively. The survivors were recovered by plating on TH agar. Survival rate was calculated as the CFU mL^−1^ at a given time divided by the CFU mL^−1^ at time zero. Values marked with an asterisk are statistically significantly different between groups (*P* < 0.05, ANOVA comparison for all pairs using Tukey-Kramer HSD).

Our transcriptome analysis suggested that the accumulation of protons by surface associated insoluble glucans and the resultant pH drop on cell surface could allow *S. mutans* to pre-condition itself against acid-stress. To test this, UA140 wild-type and *gtfBC* deficient strain were cultivated in buffered (pH7.5) MDM supplemented with either sucrose or glucose. Cells were collected and their survival rates were compared after being exposed to the medium with a lethal pH (pH 2.8) for 0.5 h and 1 h. As shown in Fig. 4, exposure of *S. mutans* wild-type cells pre-grown in the presence of sucrose to pH 2.8 for 0.5 h did not greatly affect cell viability; however, the survival rate of cells pre-grown in the presence of glucose decreased dramatically to 0.52% (p < 0.05). After 1 h acid exposure, the difference in the survival rate between *S. mutans* cells pre-grown in the presence and absence of sucrose became even more drastic (20.38% vs. 0.002%) (p < 0.05). The significantly enhanced resistance to acid killing induced by sucrose suggested that sucrose metabolism plays a role in *S. mutans’* acid resistance.

Furthermore, compared to wild-type, the *gtfBC* mutant defective in synthesizing insoluble glucans suffered significantly more viability loss (p < 0.05) when exposed to the lethal pH (with survival rate of 12.67% and 0.0005% after 0.5 h and 1 h exposure, respectively) even after being pre-grown in the presence of sucrose (Fig. 4). The results further demonstrate the important role of insoluble glucans in linking *S. mutans’* acidogenicity and aciduricity.

## Discussion

Insoluble glucan production has been considered an important virulence factor of *S. mutans*, largely due to its ability to facilitate cell adherence and biofilm formation^14^,^18^. In this study, we demonstrated for the first time that insoluble glucans also play a crucial role in acid tolerance response by linking acidogenicity and aciduricity, two of the most significant physiological features of *S. mutans* relevant to its cariogenicity.

Previous studies have revealed the spatial distribution of pH within biofilms. Using a fluorescent pH indicator incorporated into the biofilm matrix, Xiao *et al.* detected low-pH microenvironments in the interior of the EPS-microcolony complexes and at the microcolony/sHA (saliva-coated hydroxyapatite) interface^22^. Our recent study corroborated these observations and further linked the pH heterogeneity of *S. mutans* microcolonies to the difference in the metabolic activity of the microcolonies^20^. Based on the *S. mutans* cell surface pH analysis, we speculated that insoluble glucans within *S. mutans* microcolonies might accumulate protons from its own acid production to create localized acidic microenvironment within the biofilms. In this study, we tested our hypothesis that *S. mutans* retains protons through surface-associated glucans and this results in up-regulation of acid tolerance response and leads to acid preconditioning for combating future acid challenge.

By measuring the change in fluorescence intensity of the surface-expressed pH-sensitive pHluorin, we revealed a significant positive correlation between sucrose-availability/GtfBC-activity and the cell’s ability to achieve pH drop on its surface when they were switched from neutral to acidic pH (Fig. 1). By performing an *in vivo* (cell-surface associated) and *in vitro* (enzymatically prepared) insoluble glucans pHrodo Green staining assay (Fig. 2), we confirmed that the surface component in *S. mutans* involved in protons recruitment is indeed the insoluble glucans synthesized by GtfBC enzyme.

Although the exact mechanism of proton accumulation is not known at this time, a few lines of evidence suggested that the ability of glucans to accumulate protons could be due to their chemical as well as physical properties. Insoluble glucans might chemically bind and recruit protons from its surroundings to the cell surface due to its negatively charged attribute. Employing a polarization circuit through platinum electrodes exposed *in S. mutans* suspension, Yamashita *et al.* showed that the number of cells adsorbed to the anode were much greater than the number of cells adsorbed to the cathode, indicating the cell surfaces of *S. mutans* are generally negatively charged^23^. Meanwhile, glucans-coated *S. mutans* cells adhered to positively charged DEAE-Sephadex, but not to uncharged Sephadex, suggesting the cell surface-adsorbed glucans contributed to the net negative charge on *S. mutans* cell surface^23^. The negatively charged nature of insoluble glucans could be attributed to the presence of acidic groups, e.g. phosphate or sulphate groups on the polysaccharides^24^. Meanwhile, the incorporation of lipoteichoic acid to glucans could also contribute to the negatively charged surface^25^. Furthermore, insoluble glucans could also act as ion exchanger in restricting the diffusion of a variety of molecules and then influence the pH distribution in biofilms^22^.

In addition to chemical binding, the accumulation of proton on the cell surface could also be achieved through physical entrapment. Insoluble glucans produced by GtfB composes mostly of α-(1, 3)-linked glucose moieties; while GtfC synthesizes a polymer with both α-(1, 3)-linked and α-(1, 6)-linked glucans. There is a marked increase in the number of α-(1, 3)-linkages and a higher percentage of 3-linker branch points in the surface-formed glucans compared with that formed in solution^12^. The molecular structure of insoluble glucans could decrease the porosity of the biofilm matrix, thus producing a strong sieving effect to physically “trap” proton-binding macromolecules^19^.

The “trapping” of proton by surface-associated insoluble glucans and the resultant pH drop in the cell surface could potentially triggers acid-adaptive response and allows *S. mutans* to prepare itself for further acid insult. Using global transcriptomic analyses, we revealed a set of genes encoding diverse functions in multiple cellular pathways whose expression was differently regulated by pH. Many of these genes are acid-inducible and, more importantly, the up-regulation of these genes under acidic condition is also dependent on the presence of insoluble glucans (Fig. 3). Further analysis assigned many of these genes to one of the following four previously proposed major mechanisms of acid tolerance in *S. mutans*:

1. Maintenance of internal pH homeostasis via proton pumping or intracellular proton consumption: Our data revealed F_1_F_0_-H/ATPase-encoding genes as one of the acid-induced operons which was clearly impacted by the loss of glucans (Fig. 3). In *S. mutans*, the membrane-bound, acid-stable, proton-translocating F_1_F_0_-H/ATPase is largely responsible for the maintenance of internal pH homeostasis, and plays an important role in acid tolerance^7^,^8^. Furthermore, we observed the induction of genes encoding enzymes involved in citrate metabolism, such as citrate synthase (citZ) and aconitate hydratase (citB). The citrate metabolism can also contribute to acid tolerance by converting citrate to oxaloacetate, which is then converted to pyruvate by oxaloacetate decarboxylase – a process that consumes an intracellular proton^9^,^26^.
2. Alteration of metabolic pathways: *S. mutans* employs the phosphoenolpyruvate:sugar phosphotransferase system (PTS)^27^ and multiple sugar metabolism (MSM) transport system^28^ for the internalization of carbohydrates. However, at acidic pH, *S. mutans* preferentially utilizes a non-PTS system^29^, such as MSM system. The MSM system is an ATP-binding cassette transporter involved in the uptake and metabolism of a wide range of sugars, including trisaccharides, disaccharides and monosaccharides^30^. Our finding is consistent with these reports, showing an up-regulated expression of msm operon when cells were exposed to low pH (Fig. 3). Our data implied that even under acidic microenvironment, *S. mutans* could still maintain its dietary carbohydrates in-take, and stay competitive within multispecies biofilms.
3. Protection and/or repair of macromolecules. A number of chaperon proteins displayed increased expression when cells encountered low pH, including GrpE, dnaK and dnaJ (Fig. 3). These chaperons intervene in a variety of stresses, including acid stress, for functions such as protein folding, re-naturation, protection of denatured proteins and evacuation of damaged ones^5^.
4. Cell-membrane change. Our study also revealed that when exposed to low pH, compared to wildtype, *gtfBC* mutant display an overall reduced expression of *fab* operon (although not statistically significant), which encodes membrane fatty acid biosynthesis function. The cell membrane fatty acid composition has been implicated to affect membrane proton permeability directly by changing the permeability of lipid bilayers to protons or indirectly by affecting the activity of the F1F0-ATPase^31^. Our resulted suggest the loss of insoluble glucans also impacts the regulation of membrane fatty acid synthesis during acid stress response.

In addition, transcriptomic analysis revealed insoluble-glucan dependent up-regulation of *gtfBC* under low pH condition, which would allow *S. mutans* to continuously produce insoluble glucans for biofilm accretion even under acidic environment. The exposure of *S. mutans* to sucrose and acidic environment has been shown to induce the expression of the *gtfBC* when growing under biofilm conditions^21^,^32^,^33^,^34^. While our results were consistent with above findings they contrasted with other reports where the expression of *gtfBC* was either reduced or remained unchanged when grown in the presence of sucrose^35^. The expression of *gtfBC* has been shown to be affected by multiple factors, including different bacteria strains, growth phase and mode, as well as environmental conditions such as medium pH, time of cultivation, and sugar concentration^21^,^32^,^33^,^34^,^35^. The observed discrepancy could potentially result from the combination of these factors.

The most intriguing finding of our transcriptomic and qRT-PCR analysis was that the induction of ATR genes is both low-pH and sucrose/glucan-dependent; while acidic environment alone was not sufficient to trigger their up-regulated gene expression as indicated by the lack of response in these gene sets when glucose was the only sugar available (Fig. 3). The transcriptome and acid killing data (Fig. 4) suggested that the insoluble glucans play a crucial role in *S. mutans’* acid resistance, likely through trapping of protons which could allow *S. mutans* to achieve preconditioning against acid stress. These results further implicate a complex interplay between insoluble glucan biosynthesis, acid production and acid tolerance.

**Figure 5:**
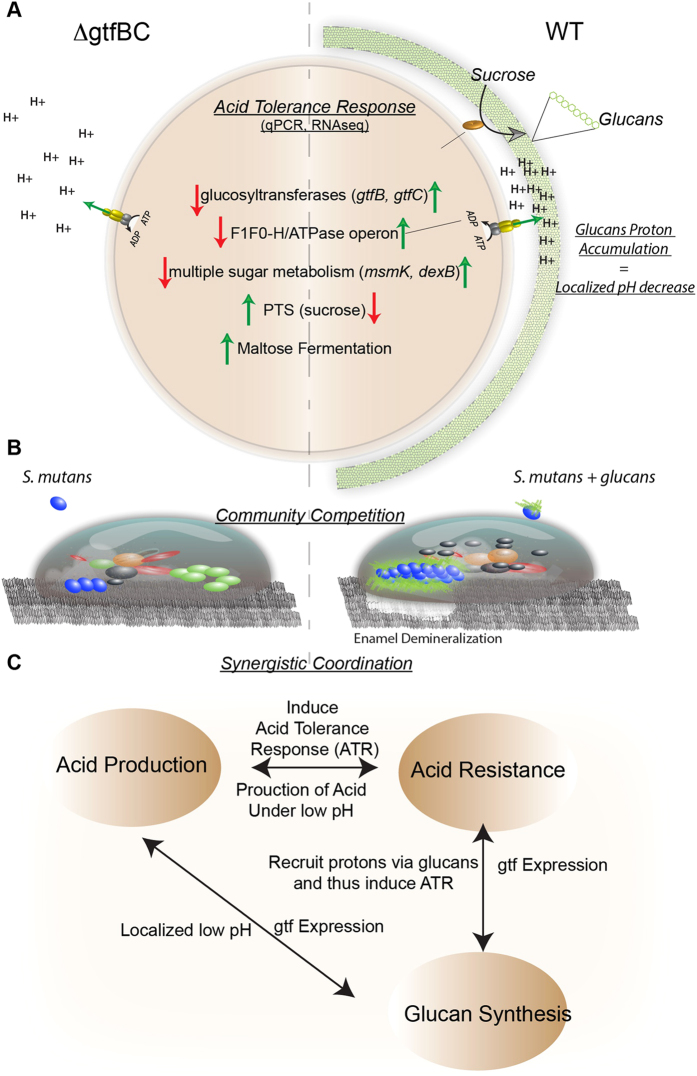
Illustration of qRT-PCR and transcriptomic data comparing gtfBC deletion and WT revealing insoluble glucan linking acidogenicity and aciduricity in S. mutans. (**A**) *S. mutans* establishes low localized pH via its surface-associated glucans and that low surface pH induces protective mechanisms for coping with acid-stress. The normal adaptive Acid Tolerance Responses (ATR) genes in WT are not induced when glucans are missing. (**B**) Illustration of how the lack of acid tolerance is likely to impact the overall persistence and growth of S. mutans within the community and (**C**) a model of how the virulence properties could be linked.

In conclusion, the results presented here deepen the understanding of the roles insoluble glucans play in the cariogenesis of *S. mutans* (summarized in Fig. 5). In addition to promoting *S. mutans* attachment and biofilm formation, our study revealed another important biological function of insoluble glucans produced by *S. mutans*. Our data suggested that *S. mutans* could retain protons from its own acid production via the surface-associated insoluble glucans. The resultant pH drop in the cell surface likely triggers acid-adaptive response and allows *S. mutans* to prepare itself for further acid insult ahead of other oral bacteria; while the absence of glucans could render cells to the instant acidic attack with detrimental effect. It has been postulated that pre-stress of bacteria within a biofilm community provides an insurance policy against future acid challenges, with ATR predicted to be the key cellular response for bacteria survival^23^. Meanwhile, the significantly enhanced resistance to acid killing induced by sucrose indicated that sucrose metabolism itself plays a role in *S. mutans’* acid resistance. Sucrose utilization enables *S. mutans* to also survive acid stress better than many oral bacteria since it results in the synthesis of these glucans. It is tempting to speculate that this is probably the link between *S. mutans* becoming a major pathogen when refined sugar was introduced. Recently the evolution of these genes was investigated revealing a possible intra-genomic gene duplication event after the initial gene acquisition from other genera, which they suggest could conceivably be linked to selection pressure such as consumption of refined sugar^36^. Although that work was focused on GTFs and their role in biofilm formation, we show that it also allows *S. mutans* to achieve a low pH growth advantage. Our study demonstrated the positive correlation between sucrose-mediated glucans biosynthesis and the acid tolerance. More importantly, we revealed the crucial role of the insoluble glucans in linking acid production and acid tolerance, the two most important cariogenic factors relevant to cariogenesis of *S. mutans.*

### Experimental Procedures

#### Bacterial strains and growth conditions

*S. mutans* UA140 wild type, the UA140 *gtfBC*-deficient strain (kindly provided by H. Kuramitsu, University of Buffalo, NY, USA), as well as UA140 pHluorin-SpaP fusion strain^20^ and its corresponding *gtfBC*-deficient derivative were cultured in Todd-Hewitt (TH) media (Difco) or MDM^37^ at 37 °C in the presence of 5% CO_2_. The *S. mutans* expressing the pHluorin-SpaP fusion and the *gtfBC*-deficient strain were cultured in the same medium supplemented with 800 μg/mL spectinomycin and 15 μg/mL erythromycin, respectively (Table 1).

### Strain construction

*S. mutans* strain O87 with cell surface-displayed ecliptic pHluorin has been constructed previously^20^. Similar method was used for generating *S. mutans* strain gtfBC/O87, a UA140 *gtfBC* derivative carrying pHluorin on its cell surface.

### Preparation of different *S. mutans* stains for fluorescence measurement/acid killing assay/transcriptome analysis

Overnight cultures (OD 600 nm = 1) of *S. mutans* wild-type and *gtfBC*-deficient strain grown in TH were diluted 1:100 in buffered (pH7.5) MDM supplemented with sucrose or glucose (20 mM)^38^. Two mL of bacterial suspension was added to the well of a 6-well flat-bottomed polystyrene microtiter plate (Corning, New York, NY) and incubated for 12 h at 37 °C in the presence of 5% CO_2_. After 12 h incubation, wild type *S. mutans* forms noticeable biofilms at the bottom of the well when sucrose was supplemented; while the rest of wells (with glucose supplement) showed bacterial growth but no significant biofilm formation. Six-well plates were centrifuged, the cells were either subjected to different pH treatment for fluorescence measurement, or collected for further acid killing assay. Alternatively, cells were washed with PBS and incubated in fresh sucrose or glucose-supplemented (20 mM) buffered MDM (phosphate buffer for the pH control; pH 7.5 or 5.5) for another 3 h, before they were harvested and processed for transcriptome analysis.

### Fluorescence measurement

Our recent study showed that under biofilm growth conditions, *S. mutans* O87 cells responded to the shift in external pH from 7.5 to 5.5 with a drastic reduction (about 70%) in fluorescence intensity within 10 min, and the signal was almost fully restored ( > 90%) after 10 min exposure to a re-adjusted external pH of 7.5. To monitor the pH change on the cell surface, *S. mutans* strain O87 and *gtfBC*/O87 were pre-grown in buffered minimal defined medium (MDM) (pH 7.5) in the presence of sucrose or glucose as described above. Cells were then switched to buffers with lower pH values (pH 7.0, 6.5, 6.0 and 5.5) and their surface fluorescence intensity was monitored after 10 minutes’ incubation. The phosphate buffer solutions were prepared by mixing potassium phosphate monobasic anhydrous and sodium phosphate dibasic heptahydrate in different ratios to obtain the desired pH value. The pH value of the buffer solutions was determined using an AB15 pH meter (Fisher). The pHluorin fluorescence signal intensity of cells was measured at different pH values by a Cary Eclipse fluorescence spectrophotometer (Varian, Mulgrave, Victoria, Australia) using a bandpass filter with a center wavelength of 400 ± 5 nm. Meanwhile, viability counting (CFU/ml) on agar plates was performed for each sample and was factored in later to normalize the cell biomass.

### Acid killing assay

To test their acid resistance ability, *S. mutans* wild-type and *gtfBC*-deficient strains were pre-grown as described above. Cells were immediately subjected to acid stress by being incubated in one mL of pH 2.8 glycine solution for 0, 0.5 h and 1 h^39^. The killing process was terminated by washing cells with one mL of PBS for three times. Collected cells were passed through a 26 gauge, 5/8 inches (15.9 mm) long needle 10 times to break cell clumps before being plated on non-selective agar plates. Plates were incubated at 37 °C and colonies were counted after 3 days. The acid resistance of different *S. mutans* strains was expressed as the survival rate of bacterial cells after being exposed to lethal pH, which was calculated as the percentage of number of surviving cells to the total initial cells (as the CFU mL^−1^) before the treatment.

### Preparation of insoluble glucans

*S. mutans* strains were grown in TH medium at 37 °C for 16 h in the presence of 5% CO_2_. The cell-free GTF enzymes were prepared according to the method by Ebisu *et al.*^40^. The prepared enzymes were incubated with 100 mM sucrose in 0.1 M phosphate buffer, pH 6.8, at 37 °C for 48 h. The insoluble polysaccharide formed in the reaction mixture was collected by centrifugation at 45,000× g for 30 min and washed three times with excess distilled water. The total carbohydrate was estimated by the phenol sulphuric acid method^41^, and glucose was used as standard.

### Confocal laser scanning microscope imaging

*S. mutans* UA 140 were grown in the MDM (buffered to pH 7.0) supplemented with glucose or sucrose (20 mM) for 16 h. The cells were collected by centrifugation and washed twice with PBS. Then the *S. mutans* cells and insoluble glucans prepared *in vitro* were spread onto cover glass (Fisher). To prevent glucans from detaching from cover glass, 1% agarose dissolved into phosphate buffer (pH 7.0 or 5.5) was poured onto the surface of glucans pellets. For visualization, 40 μl of phosphate buffer solution (pH 7.0 or 5.5) containing 40 μg/mL pHrodo Green STP Ester dye (Lifetech), which displays enhanced fluorescence intensity with the drop in pH, was added to the pellets and sample was incubated at room temperature for 30 min before imaging.

All images were collected with a Zeiss LSM 5 PASCAL confocal laser scanning microscope (CLSM) using LSM 5 PASCAL software (Zeiss, Jena, Germany). Excitation at 488 nm with an argon laser in combination with a 505 nm longpass emission filter was used for pH fluorescence imaging. The scanning module of the system was mounted onto an inverted microscope (Axiovert 200M). The 40 × (NA/1.3) numerical aperture oil-immersion objectives were used for imaging. Image stacks (1024 by 1024-pixel tagged image file format) were taken.

### mRNA isolation and sequencing

*S. mutans* wild-type and *gtfBC*-deficient strain incubated under buffered pH 7.5 or 5.5 condition for 3 h in the presence of sucrose or glucose were collected by centrifugation as described and then frozen in liquid nitrogen. Total RNA extraction and purification was performed using the mirVana RNA extraction kit (Life Technologies) and the RNA Clean/Concentrator™ kit (Zymo Research, Irvine, CA). DNA was removed from the samples by adding 1 μl (2U) Turbo™ DNAse (Life Technologies) and incubating samples at 37 °C for 30 min. After DNA removal, 16S rDNA PCR was performed using the same protocol and primers as described in McLean *et al.*^42^ to verify that DNA was removed. To remove rRNA in total RNA extracts the RiboZero™ Magnetic Kit (Epicenter) was used according to manufacturer’s instruction. mRNA was purified by using the Zymo RNA Clean and Concentrator™ kit (Zymo Research). RNA concentration and integrity was monitored before and after rRNA removal by using the Agilent RNA 6000 Nano Kit (Agilent Technologies, Inc. Santa Clara, CA) and the Agilent RNA 6000 Pico Kit (Agilent Technologies), respectively. cDNA library from rRNA-depleted RNA was generated by using random-primed cDNA synthesis methods according to the ScriptSeq™ v2 RNA-Seq Library Preparation Protocol (Epicenter). Prior to second strand cDNA synthesis the di-tagged cDNA was purified using the Agencourt AMPure XP system (Beckman Coulter). Index-reads supplied with the ScriptSeq Kit were added to the libraries which then were PCR amplified for 15 cycles. RNA-Seq libraries were purified and quantified by using the Agencourt AMPure XP system (Beckman Coulter) and the Agilent DNA 1000 protocol (Agilent Technologies), respectively. Sequencing of cDNA libraries was performed by using an Illumina HiSeq 2000 platform (100 bp paired end reads) which has the capacity of 19 GB per lane providing high coverage of reads. Sequencing was carried out at the JCVI sequencing facility JTC. cDNA sample concentrations were normalized at JTC prior to sequencing. Using each sample’s individual barcodes, the Illumina data was deconvolved into the respective samples. After trimming the bar codes, low-quality and short sequences (<100 bp) were removed by using the CLC Genomics Workbench Software (CLCbio, Aahus, Denmark). The following CLC-parameters were applied during paired read sequence trimming and quality control: quality score setting: NCBI/Sanger or Illumina Pipeline 1.8 and later, minimum distance: 180, maximum distance: 250.

### Read mapping of raw cDNA reads onto *S. mutans* 159 genome

Expression values for each mRNA sample were generated by BWA mapping^43^ of both filtered fragment and paired reads onto the well-annotated reference genome *S. mutans* UA159. Reads were mapped with the default BWA option (96% sequence identity). CLC RNAseq plugin software was used to normalize and determine statistical significance of expression. DESeq, which uses a model based on the negative binomial distribution with variance and mean linked by local regression^44^ was also employed to validate the expression observed with good agreement between the methods. Additional investigations of the transcription start sites, co-expressed genes, predicted small RNAs and normalized expression using by upper quartile normalization were performed with Rockhopper^45^ and manual curation. Genes with log_2_ 1.5 fold changes in the WT or gtfBC mutant at pH 5.5 relative to pH 7.5 or between WT and gtfBC mutant at pH 5.5 are provided as Supplementary material (Fig. S1-S6).

### Real-time quantitative PCR

Total RNA was extracted and purified according to above method. The RNA concentration was determined spectrophotometrically using a Nanodrop instrument (Nanodrop 2000; Thermo Scientific). The integrity of the RNA was assessed by agarose gel electrophoresis. cDNAs of *S. mutans* wild-type and *gtfBC*-deficient strain were synthesized according to the protocol of Transcriptor first strand cDNA sysnthesis kit (Roche).

Quantitative real-time PCR was performed using the iQ SYBR Green supermix (Bio-Rad Laboratories) on a Bio-Rad iQ5 real-time PCR detection system (Bio-Rad Laboratories, Inc., CA). The quantitative real-time PCR reaction mixture (20 μl) contained 1 × iQ SYBR Green supermix, 0.1μg cDNA, and 0.5 μM of the appropriate forward and reverse PCR primers (Table S1). The reactions were incubated at an initial denaturation at 95 °C for 10 min, followed by a 40-cycle amplification consisting of denaturation at 95 °C for 15 s, annealing at 55 °C for 15 s, and extension at 72 °C for 15 s. All primers pairs were checked for primer-dimer formation by using the dissociation curve analysis. A standard curve was plotted for each primer set with C_t_ values obtained from amplification of known quantities of *S. mutans* cDNAs. The expression level of *gtfB*, *gtfC*, *atpA*, *atpD*, *msmK*, and *dexB* gene was normalized using the *S. mutans* 16S rRNA gene, and the acid-induced fold changes of the expression levels in *S. mutans* wild-type or its *gtfBC* derivative were calculated by dividing the expression at pH 5.5 by that at pH 7.5. There was no significant difference in the expression of the 16S rRNA gene under the various conditions (data not shown). Each assay was performed with at least two independent RNA samples in triplicates.

## Additional Information

**How to cite this article**: Guo, L. *et al.* The well-coordinated linkage between acidogenicity and aciduricity via insoluble glucans on the surface of *Streptococcus mutans*. *Sci. Rep.* **5**, 18015; doi: 10.1038/srep18015 (2015).

## Supplementary Material

Supplementary Information

## Figures and Tables

**Table 1:** Strains used in this study.

Strain	Relevant characteristics	Reference
*S. mutans* UA140	Wild-type *S. mutans* Kan^s^ Erm^s^	46
*S. mutans* strain O87	UA140::Φ(*ldh*_*p*_-leading sequence-*gfp*-linker-*spaP*), Spec^r^	20
*S. mutans* strain *gtfBC*	UA140i*gtfBC*, Erm^r^	47
*S. mutans* strain *gtfBC*/O87	UA140::Φ:*ldhp*-leading sequence-*gfp*-linker-*spaP*)Δ*gtfBC*,Spec^r^ Erm^r^	This work
