# Supplementary material for: The well-coordinated linkage between acidogenicity and aciduricity via insoluble glucans on the surface of Streptococcus mutans
Source: Sci Rep. 2015 Dec 10;5:18015. doi: 10.1038/srep18015 (PMC4675080; doi:10.1038/srep18015)
Supplement: Supplementary Information [file srep18015-s1.pdf]

Supplementary Information for

The well-coordinated linkage between acidogenicity and aciduricity via insoluble glucans on the surface of *Streptococcus mutans*

Lihong Guo, Jeffrey S. McLean, Renate Lux, Xuesong He[‡] and Wenyuan Shi[‡]

[‡]Corresponding authors

Email: xhe@ucla.edu, wshi@dentistry.ucla.edu

This document contains:

1) **Supplementary Figures: Fig. S1-S6**
2) **Supplementary Tables: Table S1-S3**

1) Supplementary Figures:

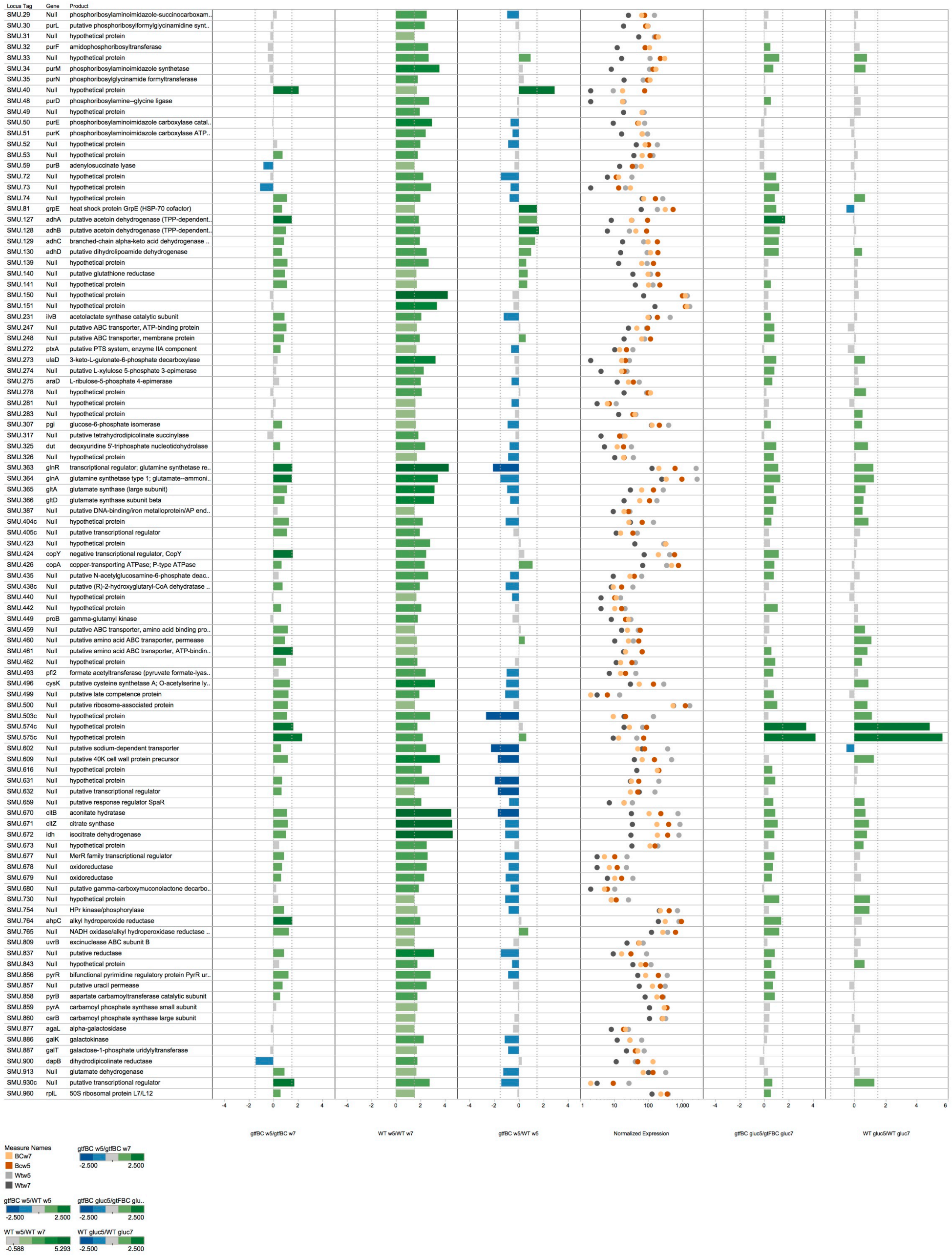

Fig. S1: Genes with log_2 1.5 fold increase in the WT pH 5.5 relative to pH 7.5. Dashed vertical lines indicate significant fold change. Colored bars indicate log_2 fold changes for differential expression. Filled colored circles indicate normalized expression values for each condition tested with sucrose. gtfBC mutant with sucrose pH 5.5 (bcw5), gtfBC mutant with sucrose pH 7.5 (bcw7), WT with sucrose pH 5.5 (Wtw5), WT with sucrose pH 7.5 (wtw7.5).

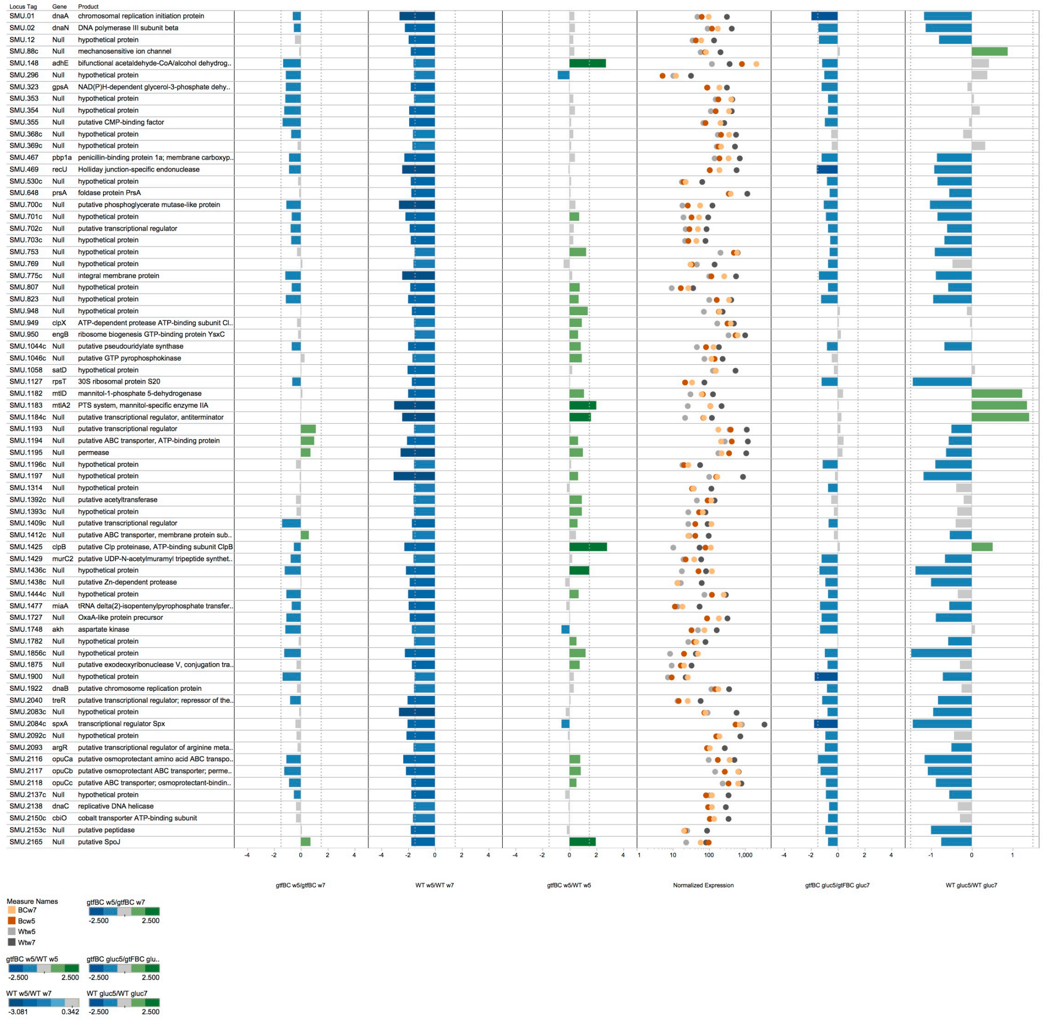

Fig. S2. Genes with greater than log_2 1.5 fold decrease in the WT at pH 5.5 relative to pH 7.5. Dashed vertical lines indicate significant fold change. Colored bars indicate log_2 fold changes for differential expression. Filled colored circles indicate normalized expression values for each condition tested with sucrose. gtfBC mutant with sucrose pH 5.5 (bcw5), gtfBC mutant with sucrose pH 7.5 (bcw7), WT with sucrose pH 5.5 (Wtw5), WT with sucrose pH 7.5 (Wtw7).

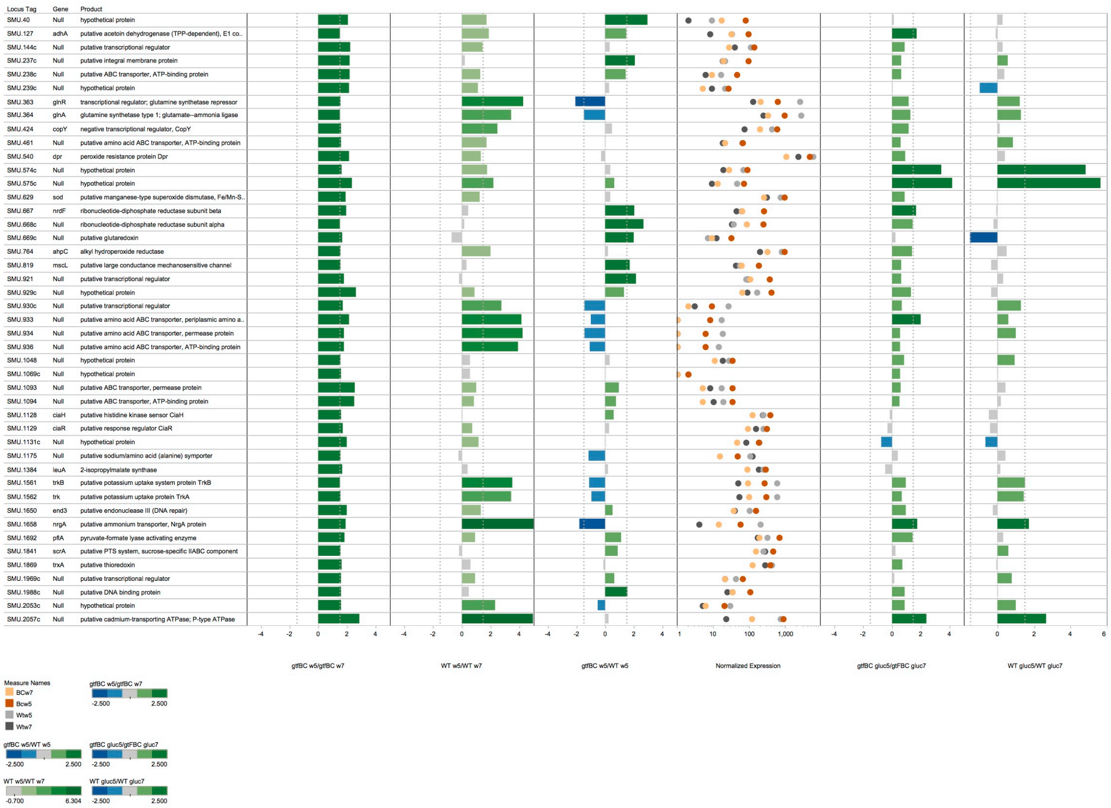

Fig. S3: Genes with log_2 1.5 fold increase in the gtfBC mutant at pH 5.5 relative to pH 7.5. Dashed vertical lines indicate significant fold change. Colored bars indicate log_2 fold changes for differential expression. Filled colored circles indicate normalized expression values for each condition tested with sucrose. gtfBC mutant with sucrose pH 5.5 (bcw5), gtfBC mutant with sucrose pH 7.5 (bcw7), WT with sucrose pH 5.5 (Wtw5), WT with sucrose pH 7.5 (wtw7.5).

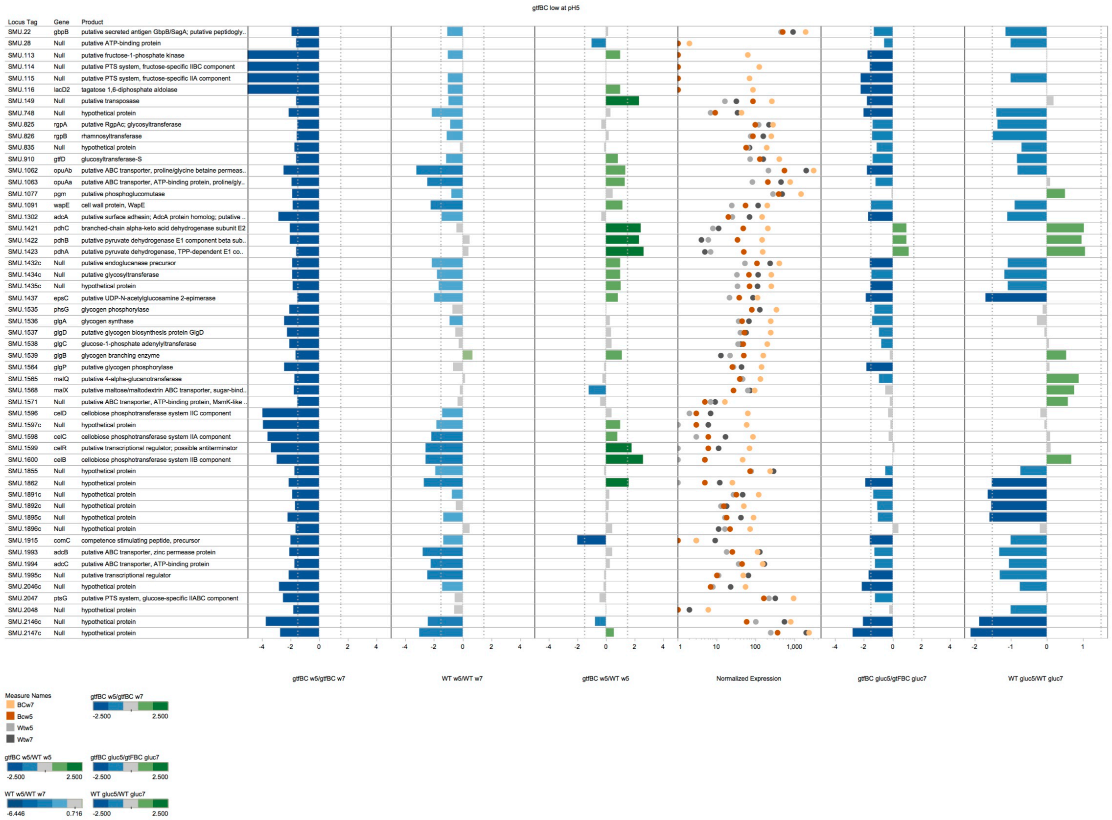

Fig. S4: Genes with log_2 1.5 fold decrease in the gtfBC mutant at pH 5.5 relative to pH 7.5. Dashed vertical lines indicate significant fold change. Colored bars indicate log_2 fold changes for differential expression. Filled colored circles indicate normalized expression values for each condition tested with sucrose. gtfBC mutant with sucrose pH 5.5 (bcw5), gtfBC mutant with sucrose pH 7.5 (bcw7), WT with sucrose pH 5.5 (Wtw5), WT with sucrose pH 7.5 (wtw7.5).

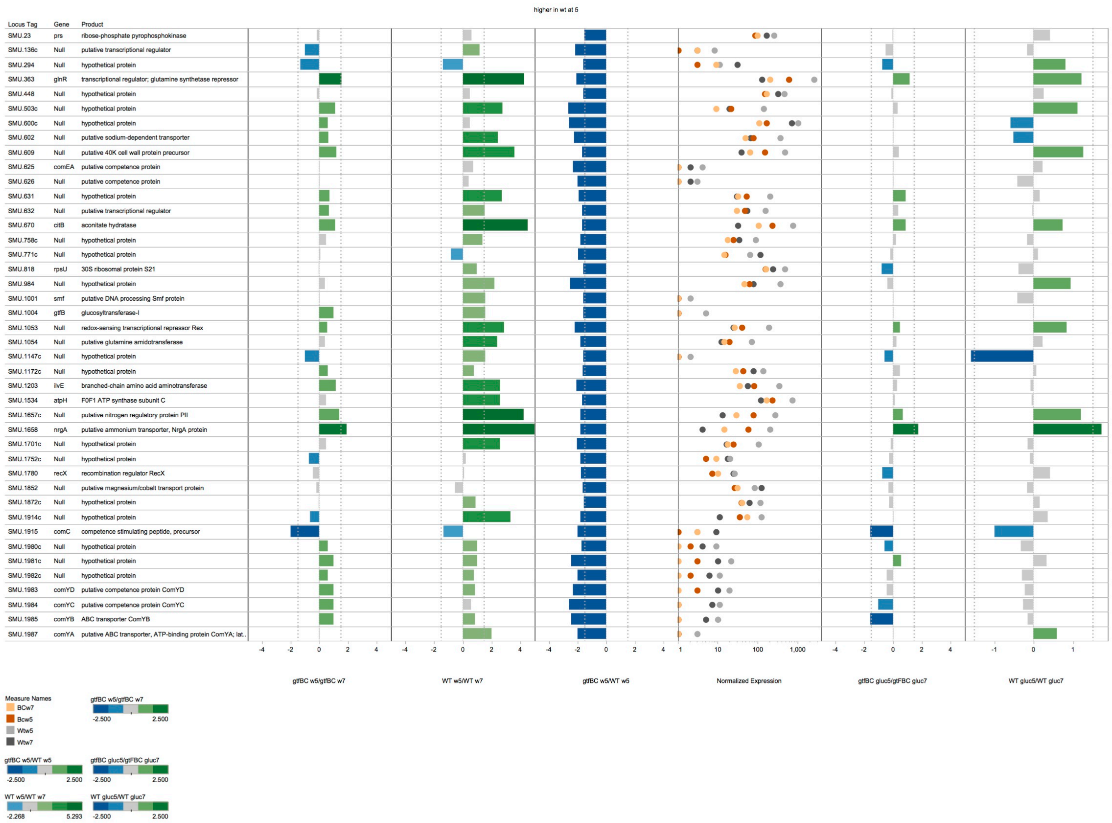

Fig. S5: Genes with $\log_2$ 1.5 fold increase in the WT at pH 5.5 relative to gtfBC mutant pH 5.5. Dashed vertical lines indicate significant fold change. Colored bars indicate $\log_2$ fold changes for differential expression. Filled colored circles indicate normalized expression values for each condition tested with sucrose. gtfBC mutant with sucrose pH 5.5 (bcw5), gtfBC mutant with sucrose pH 7.5 (bcw7), WT with sucrose pH 5.5 (Wtw5), WT with sucrose pH 7.5 (wtw7.5).

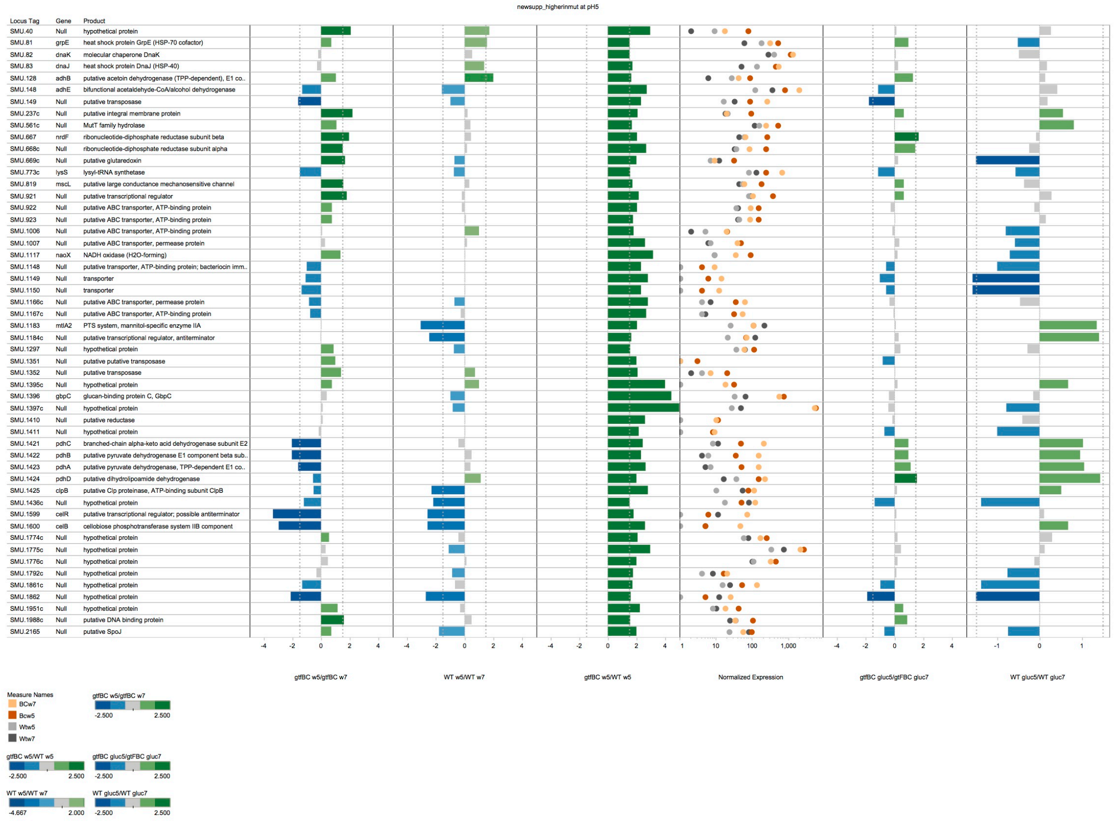

Fig. S6: Genes with log_2 1.5 fold decrease in the WT at pH 5.5 relative to gtfBC mutant pH 5.5. Dashed vertical lines indicate significant fold change. Colored bars indicate log_2 fold changes for differential expression. Filled colored circles indicate normalized expression values for each condition tested with sucrose. gtfBC mutant with sucrose pH 5.5 (bcw5), gtfBC mutant with sucrose pH 7.5 (bcw7), WT with sucrose pH 5.5 (Wtw5), WT with sucrose pH 7.5 (wtw7.5).

2) Supplementary Tables:

S1 Table. Primers used in this study

Gene name	Primer sequence (5’-3’, forward and reverse)
gtfB	AGCAATGCAGCCAATCTACAAAT
	ACGAACTTTGCCGTTATTGTCA
gtfC	GGTTTAACGTCAAAATTAGCTGTATTAGC
	CTCAACCAACCGCCACTGTT
atpA	TATTGCTCGTGCTTGCGGAC
	TTTCACCCAGACCATCAACAGG
atpD	GGCGACAAGTCTCAAAGAATTG
	AACCATCAGTTGACTCCATAGC
msmK	CCTTTATATTGATGATAAACTCA
	CATATTTTCATAAACGCTCAT
dexB	AATGGCAGACTGAGTTGGA
	CACGCATAAGGTGAAGAAG
16S rRNA	CTTACCAGGTCTTGACATCCCG
	ACCCAACATCTCACGACACGAG

S2 Table. The number of differentially regulated genes in *S. mutans* wt and *gtfBC* under different conditions.

	# genes up-regulated	# genes down-regulated
[a]wt sucrose pH5.5/wt sucrose pH7	192	96
[b]gtfBC sucrose pH5.5/ gtfBC sucrose pH7	47	57
[c]gtfBC glucose pH5.5/ gtfBC glucose pH7	11	31
[d]wt glucose pH5.5/ wt glucose pH7	14	17

[a] wild type *S. mutans* cells pre-grown in the presence of sucrose were subjected to 3-hr exposure to buffered (pH5.5 and pH7)MDM medium;
[b] gtfBC mutant cells pre-grown in the presence of sucrose were subjected to 3hr exposure to buffered (pH5.5 and pH7) MDM medium;
[c] gtfBC mutant cells pre-grown in the presence of glucose were subjected to 3-hr exposure to buffered (pH5.5 and pH7)MDM medium;
[d] wild type *S. mutans* cells pre-grown in the presence of glucose were subjected to 3hr exposure to buffered (pH5.5 and pH7) MDM medium;

S3 Table. qPCR analysis of relative expression of selected genes in *S. mutans* wild type and *gtfBC*-deficient mutant grown at neutral or acidic pH in the presence of sucrose or glucose.

Functional class annotation	Gene Name	Sugar added	Wild-type Fold change (pH 5.5 vs pH7.5)[a] qPCR (avg ± SD)	*gtfBC* mutant Fold change (pH5.5 vs pH7.5)[a] qPCR (avg ± SD)
Insoluble glucans synthesis	*gtfB*	sucrose	2.63±0.55	
		glucose	0.83±0.22	
Insoluble glucans synthesis	*gtfC*	sucrose	2.27±0.71	
		glucose	0.46±0.13	
F_1F_0-H/ATPase	*atp* operon	sucrose	*atp*A:2.61±1.27<br>*atp*D:8.10±2.56	*atp*A:1.02±0.68<br>*atp*D:1.00±0.16
		glucose	*atp*A:1.51±0.31<br>*atp*D:1.07±0.21	*atp*A:0.95±0.53<br>*atp*D:0.88±0.22
Multiple-sugar metabolism (msm)	*msmK*	sucrose	4.75±1.44	-2.64±0.89
		glucose	1.71±0.15	-3.16±1.45
	dexB	sucrose	3.42±1.69	-3.52±1.71
		glucose	1.87±0.42	-2.67±0.42

[a] Normalized expression was calculated by dividing the expression in *S. mutans* at pH 5.5 by the expression at pH 7.5.
